# Elevated CO_2_ Modulates Plant Hydraulic Conductance Through Regulation of PIPs Under Progressive Soil Drying in Tomato Plants

**DOI:** 10.3389/fpls.2021.666066

**Published:** 2021-05-28

**Authors:** Shenglan Li, Liang Fang, Josefine Nymark Hegelund, Fulai Liu

**Affiliations:** Department of Plant and Environmental Sciences, Faculty of Science, University of Copenhagen, Taastrup, Denmark

**Keywords:** elevated CO_2_, abscisic acid, drought stress, plant hydraulic conductance, OPEN STOMATA 1, PIPs

## Abstract

Increasing atmospheric CO_2_ concentrations accompanied by abiotic stresses challenge food production worldwide. Elevated CO_2_ (*e*[CO_2_]) affects plant water relations *via* multiple mechanisms involving abscisic acid (ABA). Here, two tomato (*Solanum lycopersicum*) genotypes, Ailsa Craig (AC) and its ABA-deficient mutant (*flacca*), were used to investigate the responses of plant hydraulic conductance to *e*[CO_2_] and drought stress. Results showed that *e*[CO_2_] decreased transpiration rate (*E*) increased plant water use efficiency only in AC, whereas it increased daily plant water consumption and osmotic adjustment in both genotypes. Compared to growth at ambient [CO_2_], AC leaf and root hydraulic conductance (*K*_leaf_ and *K*_root_) decreased at *e*[CO_2_], which coincided with the transcriptional regulations of genes of plasma membrane intrinsic proteins (PIPs) and OPEN STOMATA 1 (OST1), and these effects were attenuated in *flacca* during soil drying. Severe drought stress could override the effects of *e*[CO_2_] on plant water relation characteristics. In both genotypes, drought stress resulted in decreased *E*, *K*_leaf_, and *K*_root_ accompanied by transcriptional responses of PIPs and OST1. However, under conditions combining *e*[CO_2_] and drought, some PIPs were not responsive to drought in AC, indicating that *e*[CO_2_] might disturb ABA-mediated drought responses. These results provide some new insights into mechanisms of plant hydraulic response to drought stress in a future CO_2_-enriched environment.

## Introduction

The atmospheric carbon dioxide concentration ([CO_2_]) has been constantly increasing, and it is predicted to reach ca. 800 ppm at the end of this century (Pan et al., 2018). The elevated [CO_2_] (*e*[CO_2_]) accompanied by global warming is expected to reduce the availability of freshwater resources, resulting in more frequent drought spells (Solomon et al., 2009). On the other hand, it is well known that *e*[CO_2_] could induce stomatal closure, thus alleviating the negative effect of drought stress (van der Kooi et al., 2016). The plant hormone abscisic acid (ABA) is involved in both drought-induced and *e*[CO_2_]-induced stomatal closure in a dual way, including root-derived and foliar ABA (Tallman, 2004; Pantin et al., 2013; McAdam and Brodribb, 2018). However, to date, the drought-related physiological and molecular mechanisms involved in the regulation of plant hydraulic conductance under *e*[CO_2_] remain largely elusive.

It is widely accepted that *e*[CO_2_] can enhance plant drought tolerance. Plants benefit from *e*[CO_2_] due to an increase in photosynthetic rate, and decreases in stomatal conductance (*g*_s_) and transpiration rate (*E*), resulting in improved water use efficiency (Wullschleger et al., 2002; van der Kooi et al., 2016). Another possible mechanism is the enhanced osmotic adjustment. In many different species, plants grown under *e*[CO_2_] had lower osmotic potential and altered leaf tissue properties, including leaf size and thickness, thus could maintain favorable leaf water status as soil moisture decreased (Pritchard et al., 1999; Johnson et al., 2002; Fang et al., 2019). There is increasing evidence that the positive effect of *e*[CO_2_] on plant growth is greater under abiotic stress than under optimal conditions (Cruz et al., 2018; Uddin et al., 2018). However, studies have also shown that *e*[CO_2_] could retard stomatal response to drought stress, thus increasing plant vulnerability to severe water deficits due to impaired function of stomata (Haworth et al., 2016; Yan et al., 2017; Liu et al., 2019). Moreover, the increased leaf area of plants grown under *e*[CO_2_] might increase water consumption, leading to a fast depletion of soil water, causing severe drought stress to plants (Manea and Leishman, 2015).

Fine-tuning control of the whole-plant water relations has pivotal significance for plants surviving drought stress. Stomata regulate plant water status by controlling plant water loss such that it matches the capacity of the plant root-leaf hydraulic system to supply water to leaves for photosynthesis and transpiration (Meinzer, 2002). Previous research generally confirms that leaf hydraulic conductance (*K*_leaf_) and root hydraulic conductance (*K*_root_) can account for more than 70% of the whole hydraulic conductivity in trees, therefore water transport capacity can be quantified in terms of hydraulic properties (Domec et al., 2009). Under mild drought stress, some plant species (i.e., anisohydric species) could maintain stomatal aperture for carbon gain at the cost of dysfunction of plant hydraulics; under severe drought, strong stomatal regulation occurs in order to limit hydraulic failure (Brodribb and Holbrook, 2004; Creek et al., 2018). However, in other species (i.e., isohydric species), a quick stomatal response from an onset of drought stress can limit water loss and avoid hydraulic dysfunction (Sperry, 2004; Martin-StPaul et al., 2017). Coordination between stomata and hydraulic traits provides plants’ different drought response mechanisms.

Root-derived ABA is a long-distance stress signal, released into xylem sap and transported to leaves to regulate stomatal movements (Jiang and Hartung, 2008). However, recently this common view has been challenged by the reciprocal grafting technique, which can be used to explore the ABA biosynthesis in different plant organs (Cardoso et al., 2020). In tomato plants, under external pressure or salinity stress, it has been found that the rapid biosynthesis of ABA in leaves rather than in roots predominantly induced stomatal closure (Chen et al., 2003; Sussmilch et al., 2018). In addition, the stomatal response to changes in atmospheric CO_2_ concentration is also associated with ABA signaling (Assmann and Jegla, 2016). In tomato plants, an exponential increase in the xylem sap ABA concentration ([ABA]_xylem_) coincided with a decrease in *g*_s_ during progressive soil drying. This ABA increase was more pronounced under *e*[CO_2_], indicating that an insensitivity of stomata to [ABA]_xylem_ could exist (Yan et al., 2017; Liu et al., 2019). In the ABA-deficient mutant *flacca*, neither *g*_s_ nor [ABA]_xylem_ was influenced by *e*[CO_2_] (Wei et al., 2020), which affirmed that ABA played an important role in stomatal response to *e*[CO_2_]. It is noteworthy that ABA also alters plant hydraulics under abiotic stress, and this regulation was associated with aquaporins (AQPs) (Parent et al., 2009; Dayer et al., 2017; Rosales et al., 2019). AQPs, are water channels belonging to the Major Intrinsic Protein (MIP) superfamily, which play an important role in transport of water and other small neutral molecules across cellular membranes (Reuscher et al., 2013). The plasma membrane intrinsic proteins (PIPs), constituting the largest plant AQP subfamily, have a major role in controlling transpiration and hydraulic conductance during soil drying (Kapilan et al., 2018; Shekoofa and Sinclair, 2018). In barley, PIP2;2 and ABA were both required to enhance *K*_root_ and maintain plant water status under drought stress (Veselov et al., 2018). Earlier research generally confirms that gene expression of *PIP*s is upregulated by ABA and downregulated by severe drought stress (Mahdieh et al., 2008; Dayer et al., 2017; Zupin et al., 2017), and the magnitude of PIP regulations could be an indicator of plant drought tolerance (Grondin et al., 2015; Zupin et al., 2017). Although there is no consistent correspondence between hydraulic conductance and abundance of specific AQPs, leaf dehydration under drought stress could be due to unbalanced expression of AQPs in leaves and roots (Nada and Abogadallah, 2014).

Recently, *e*[CO_2_] has been found to regulate plant hydraulics in various species and in short-/long-term responses (Bunce, 1996; Domec et al., 2009; Hao et al., 2018; Fang et al., 2019). Negative impacts of soil water deficit on plant hydraulic properties could be alleviated by *e*[CO_2_] (Liu et al., 2019), and the reduction in hydraulic conductance under *e*[CO_2_] affected whole-plant water use by inducing a decline in *g*_s_ and *E* (Domec et al., 2009). In addition, our recent study (Fang et al., 2019) showed that *e*[CO_2_] reduced the hydraulic conductance of leaves and roots in wild-type tomato but not in the ABA-deficient mutant, which was associated with a downregulation of leaf and root PIPs in wild-type. On the contrary, a recent study on coffee plants found that *e*[CO_2_] contributed to the maintenance of the whole-plant hydraulic conductance under drought conditions, which was associated with a higher transcript abundance of most aquaporin genes (Avila et al., 2020). Therefore, to further understand the complexity of plant hydraulic responses to *e*[CO_2_] and drought stress, we need to classify different drought stress intensities and different definitions of hydraulic conductance.

OPEN STOMATA 1 kinases (OST1/SnRK2.6) has been identified to be involved in both stomatal ABA and CO_2_ signaling pathways (Shi et al., 2015), but the role of OST1 in the interaction between CO_2_/ABA signal transduction is still controversial (Takahashi et al., 2018). A common view is that OST1 is required in the ABA-induced stomatal closure acting as a positive regulator, but there are results indicating that osmotic stress could activate OST1 activity in an ABA-independent pathway (Yoshida et al., 2006). Recently, ABA was shown to amplify CO_2_ effects through the regulation of OST1 (Hsu et al., 2018), while in other situations OST1 might regulate CO_2_-induced stomatal closure in the absence of ABA (Wang et al., 2015). In the CO_2_ signaling pathway, PIP2 transports both water and CO_2_ (Mori et al., 2014). Under *e*[CO_2_], PIP2;1 in *Arabidopsis thaliana* guard cells could indirectly interact with OST1 to induce stomatal closure through increasing CO_2_ permeability (Wang et al., 2015). However, in the ABA-dependent pathway, OST1 has been shown to enhance PIP2;1 water transport activity, thus contributing to ABA-mediated regulation of hydraulic conductance (Grondin et al., 2015). These findings highlight an undescribed link between ABA and *e*[CO_2_] in regulating plant hydraulics, which merits further investigations.

The objective of the present study was to investigate the effect of CO_2_ elevation on the response of water relations and transcript levels of *PIP*s and *OST1* to progressive soil drying in two tomato genotypes differing in endogenous ABA levels. Plants were grown in two atmospheric [CO_2_] (400 and 800 ppm) environments and subjected to progressive soil drying by withholding irrigation from the pots. Plant water consumption, leaf water relations, leaf and root hydraulic conductance, [ABA]_xylem_, and transcript levels of *PIP*s and *OST1* were determined during progressive soil drying. It was hypothesized that *PIP*s and *OST1* might be involved in the *e*[CO_2_]-regulated plant hydraulic responses to soil drying.

## Materials and Methods

### Experiment Setup

A pot experiment was conducted in climate-controlled greenhouses at the Faculty of Science, University of Copenhagen, Taastrup, Denmark. Seeds of isogenic tomato cv. Ailsa Craig (AC) and an ABA-deficient tomato mutant (*flacca*; *Solanum lycopersicum*) were provided by the Lancaster Environment Centre (Lancaster University, United Kingdom). The ABA-deficient mutant is impaired in the oxidation of ABA-aldehyde to ABA, thus possessing significantly lower endogenous ABA concentrations than AC (Sagi et al., 2002). All plants were grown in 4-L pots filled with 2.6 kg of peat material (Plugg-och Såjord-Dry matter ca. 110 kg m^−3^, organic matter >95%, pH 5.5–6.5 and EC 1.5–2.5 mS cm^−1^). Four weeks after sowing, perlite was covered on the soil surface to minimize evaporation and fertilizers as NH_4_NO_3_ (2.8 g) and H_2_KPO_4_ (3.5 g) per pot were added together with irrigation water to avoid any nutrient deficiency.

From sowing, the plants were grown in two greenhouse cells with CO_2_ concentrations of 400 ppm (ambient CO_2_, *a*[CO_2_]) and 800 ppm (elevated CO_2_, *e*[CO_2_]), respectively. During the experiment, the actual daily average [CO_2_] was 420.2 and 804.2 ppm, respectively. The [CO_2_] in the cells was sustained by pure CO_2_ emission from a bottle tank and distributed evenly by the internal ventilation system. The [CO_2_] in the cells was monitored every 6 s by a CO_2_ Transmitter Series GMT220 (Vaisala Group, Helsinki, Finland). The climate conditions in the two glasshouse cells were set at: 20/16 ± 2°C day/night air temperature, 60 ± 2% relative humidity, 16 h photoperiod, and > 500 μmol m^−2^ s^−1^ photosynthetically active radiation (PAR) supplied by sunlight plus LED lamps (Philips GreenPower LED toplighting, Denmark). The vapor pressure deficit in the greenhouse cells ranged from 0.8 to 1.0 kPa.

After seedling establishment, the pots were constantly irrigated to 90% of the pot holding capacity. The soil drying treatment started at March 6 and March 15, 2018 for AC tomato and *flacca*, respectively (5 weeks after sowing). In each cell and genotype, at the onset of drought stress, four well-watered plants were harvested as the initial control; during progressive soil drying, four plants were well watered to 95% of the pot water holding capacity as control plants, and other 16 tomato plants were subjected to progressive soil drying by withholding irrigation from the pots until transpiration rate (*E*) decreased to ~10% of the control plants. The drought-stressed plants were harvested four times at different soil water statuses; and for each cell and each genotype at each harvest, four plants were harvested.

### Measurements

#### Soil Water Status

Soil water content was measured daily by weighing the pots with an Analytical Balance (Sartorius Model QA35EDE-S) at 15:30 h and expressed as the fraction of transpirable soil water (FTSW). The daily value of FTSW was estimated as the ratio between the amounts of transpirable soil water that remained in the pots and the total transpirable soil water (TTSW). TTSW was defined as the difference of pot weight between 100% water holding capacity (i.e., 4.5 kg) and when *E* of the drought-stressed plant decreased to ~10% of the control plant (i.e., 2.5 kg). Then FTSW was calculated as:

$$\mathrm{FTSW}=\left({\mathrm{WT}}_{n}-{\mathrm{WT}}_{f}\right)/\mathrm{TTSW}$$

(1)

where WT_n_ is the pot weight on a given date, WT_f_ is the pot weight at the time when *E* of the drought plant was 10% of the control plant (i.e., 2.5 kg).

#### Transpiration Rate

During progressive soil drying, instantaneous transpiration rate (*E*, mmol m^−2^ s^−1^) was measured daily on the last youngest upper canopy fully expanded leaves between 9:00 and 12:00 h with a portable photosynthetic system (LiCor-6400XT, LI-Cor, NE, United States). Measurements were performed on one leaf per plant and four biological replicates for each experimental condition and genotype. The chamber environment of measurements was as follows: 20°C cuvette temperature, 1,500 μmol m^−2^ s^−1^ PAR, and [CO_2_] of 400 ppm for *a*[CO_2_] and 800 ppm for *e*[CO_2_] growth environments, respectively. The settings in the chamber provided the optimum environment for plant transpiration; therefore, we assumed that the measured *E* at leaf scale could represent the maximum transpiration capacity in the day.

#### Plant Water Consumption and Leaf Water Relations

Daily water consumption (L) was determined by weighing the pots at 15:30 h every day. Cumulative water consumption (L) was evaluated from the beginning of drought stress to each harvest day. At each harvest, total dry weight (leaf dry weight and stem dry weight) was measured on four replicates for each experimental condition and genotype. Cumulative dry weight (g) was evaluated from the beginning of drought stress to each harvest day. The slopes of the linear relationship between cumulative water consumption and cumulative dry weight represented plant water use efficiency (WUE_plant_) and expressed as g L^−1^.

At last harvest, after determination of *E*, the same leaf was collected to measure relative water content (RWC, %) and osmotic potential (Ψ_π_, MPa) on four replicates for each experimental condition and genotype. After excision of leaves, the fresh weight (FW) was recorded immediately. Then the turgid weight (TW) was then recorded followed by a re-hydration period in distilled water for 4 h, oven-dried at 75°C for 48 h and dry weight (DW) was measured. Relative water content (RWC, %) was then calculated as:

RWC=(FW−DW)/(TW−DW) × 100

(2)

Ψ_π_ was measured using a psychrometer (C-52 sample chamber, Wescor Crop, Logan, UT, United States) connected to a microvoltmeter (HR-33T, Wescor, Logan, UT, United States) at 22 ± 1°C. Ψ_π_ at full turgor was then determined as Ψ_π_ × RWC. Osmotic adjustment (OA, MPa) was calculated as the difference in Ψ_π_ at full turgor between well-watered and drought-stressed plants.

#### Leaf and Root Hydraulic Conductance

At each harvest, leaf hydraulic conductance (*K*_leaf_, mmol m^−2^ s^−1^ MPa^−1^) was measured on four biological replicates for each experimental condition and genotype using the evaporative flux method (Sack et al., 2002; Brodribb and Holbrook, 2003; Simonin et al., 2015; Xiong et al., 2015). Following determination of *E*, four young fully expended leaves from four plants per [CO_2_] environment per genotype were excised, immediately wrapped in a plastic bag to avoid water loss, and placed in a scholander-type pressure chamber (Soil Moisture Equipment Corp., Santa Barbara, CA, United States) for determination of leaf water potential (Ψ_leaf_). Ψ_leaf_ represented the driving force, and instantaneous *E* represented the maximum water flow through the lamina. Then *K*_leaf_ was determined from the slopes of the relationship between *E* and Ψ_leaf_ measured at each harvest (Fang et al., 2019).

At each harvest, root hydraulic conductance (*K*_root_, g cm^−2^ min^−1^ MPa^−1^) was calculated on four biological replicates for each experimental condition and genotype. The whole pots were put into a scholander-type pressure chamber following the procedure described by Liu et al. (2006), then the chamber was sealed and only the above-soil part of the plants was left out. The stem was cut with a scalpel at approximately 10 cm above the soil surface. After a good seal was obtained, by pressuring the whole root system, root water potential (Ψ_root_) was determined when the xylem sap started to appear from the cutting surface. For well-watered plants, xylem sap appeared under no pressure; therefore, Ψ_root_ was equivalent to zero and was not shown here. The pressure increased at an approximate rate of 4 bar per min until it equaled Ψ_leaf_. Under such pressure, the rate of xylem sap was similar to the rate of transpiration, and the ABA concentrations in xylem sap were stable with a range of flow rate (Liang and Zhang, 1997). During the period when the pressure increased from Ψ_root_ to Ψ_leaf_, xylem sap was collected to Eppendorf tubes using a pipette, the time of sap collection was recorded and the stem cross-section area was measured. During the collecting time, we assumed that there was a linear relationship between the sap flow rate and the added pressure. Immediately after the collection, the xylem sap was weighed and then frozen in liquid nitrogen and stored at −80°C for ABA analysis. Then *K*_root_ of the whole root system was calculated following the method described by Fang et al. (2019):

$$K_{\mathrm{root}}=\frac{\mathrm{Xylem mass}}{T\times P\times S}$$

(3)

where xylem mass is the weight of the collected xylem sap (*g*); *T* is the collection time (s); *P* is the chamber pressure (MPa), which was maintained during collection; and *S* is the stem cross-section area (cm^2^). Kroot was expressed as g cm^−2^ min^−1^ MPa^−1^.

#### Xylem Sap ABA Concentrations

At each harvest, xylem sap was collected by pressurizing the potted plant in a scholander-type pressure chamber (Wei et al., 2020). Enzyme-linked immunosorbent assay was used to determine ABA concentration in xylem sap samples ([ABA]_xylem_) following the protocol of Asch (2000).

#### RNA Extraction, cDNA Synthesis, and PCR Reactions

At the last harvest, leaf (fresh and fully expanded leaf) and root (principal root) samples of four replicates were harvested at midday for each experimental condition and genotype. Samples were frozen in liquid nitrogen, stored in −80°C and later ground into powder in the presence of liquid nitrogen. RNA extractions were done from 80 to100°mg ground leaf or root material using the RNeasy Plant Mini Kit, according to the manufacturer’s instructions (Qiagen, Germany). RNA yield and purity were estimated using Nanodrop™ 1000 spectrophotometer (Thermo Fisher Scientific Inc., United States). RNA integrity was evaluated in 1% NaOCl and 1% agarose gels (Aranda et al., 2012). Purified RNA was stored at −80°C. For expression analyses, 1 μg of RNA was treated with DNase I Amplification Grade (Sigma-Aldrich, United States), and cDNA were synthesized using the iScript cDNA Synthesis Kit (Bio-Rad, United States) according to the manufacturer’s protocols. cDNA was diluted fivefold in RNase/DNase free Tris-EDTA pH 7.4 (Sigma-Aldrich) for initial tests of *PIP*s and *OST1* in reverse transcriptase PCR. To target putative water transporting plasma membrane-localized aquaporins (AQPs), the PIP subfamily was selected. Tomato-specific *PIP* primers developed previously (Reuscher et al., 2013) were used to pinpoint relevant *PIP*s in leaves and roots of this study. Tomato-specific *OST1* primers developed by Shi et al. (2015) were used to explore *OST1* expression in leaves. All initial PCR reactions using cDNA were performed using Ex taq polymerase (Takara Bio Inc., Japan) according to the manufacturer’s instructions with the addition of 2% (v/v) dimethyl sulfoxide in final reactions. PCR conditions were 94°C 4 min, 35 cycles of [30 s 94°C, 1 min 60°C, 45 s 72°C], and 7 min 72°C. Among the 12 *PIP* transcripts investigated (*PIP1;1-PIP1;3*, *PIP1;5*, *PIP1;7*, *PIP2;1*, *PIP2;4-PIP2;6*, *PIP2;8*, *PIP2;9*, and *PIP2;12*), seven *PIP*s were found to be undetectable or very low in expression levels and were not included in the subsequent quantitative PCR (qPCR) analyses. *PIP1;3*, *PIP2;1*, *PIP2;4*, *PIP2;8*, and *PIP2;9* were detected in high abundances in both leaf and root tissues, and *OST1* was also detected in high abundance in leaf tissues.

#### Quantitative Real-Time PCR Analyses

Reactions of real-time PCR (RT-qPCR) were performed using SsoAdvanced™ Universal SYBR® Green Supermix as recommended (Bio-Rad) with a CFX Connect™ RT-qPCR detection system (Bio-Rad). Analyses of optimal primer temperatures, melting curves, primer pair efficiencies, Cq values, and normalized expression (Cq) were conducted in CFX Maestro Software supplied by Bio-Rad. In addition to *PIP*s and *OST1* primer pairs, previously developed tomato-specific reference gene candidates *TIP4.1*, *SAND*, *CAC*, and *Expressed* (*EXPR*) were included in the analyses (Expósito-Rodríguez et al., 2008). *CAC* was selected as a reference gene for *PIP*s and *OST1* in RefFinder (Xie et al., 2012). Primer-specific temperatures, efficiencies, and references are available in Supporting Information Supplementary Table S1. Each treatment type was analyzed with three technical and four biological replicates. Changes to fold change less than 2-fold up or down were considered minor. The full RT-qPCR assay was conducted twice from the level of RNA extractions.

#### Statistical Analysis

The responses of *E*, *K*_leaf_, and *K*_root_ to soil drying were described by a linear-plateau model (Faralli et al., 2019):

$$\mathrm{If}\;\mathrm{FTSW}>C;\;y=y_{\mathrm{initial}}$$

(4)

$$\mathrm{If}\;\mathrm{FTSW}<C;\;y=y_{\mathrm{initial}}+S\;\times \;\left(\mathrm{FTSW}-C\right)$$

(5)

where *y* denotes *E*, *K*_leaf_, or *K*_root_, and y_initial_ denotes *E*_max_, *K*_leaf max_, or *K*_root max_; *C* denotes the FTSW threshold at which *y* started to diverge from *y*_initial_ (expressed as C_E_, C_kl_, or C_kr_, respectively). The parameters *y* and *C* were estimated by PROC NLIN of PC SAS 9.4 (SAS Institute Inc., Cary, NC, United States, 2002–2012) and the coefficient of determination (*r*^2^) was calculated. Statistical comparison of each parameter obtained from the linear-plateau regression between [CO_2_] treatments was performed by *t*-test using MedCalc statistical software 19.0.7.

Data were statistically analyzed using Microsoft Excel, SPSS 22.0 software (IBM SPSS Software, New York, United States), GraphPad Prism 8.4 software, and CFX Maestro Software (Bio-Rad). In order to compare the responses of the measured variables to soil drying, the data of daily water consumption and [ABA]_xylem_ were plotted against the FTSW such that the divergence between the variables measured on plants exposed to *a*[CO_2_] and *e*[CO_2_] at a given FTSW value could be seen, and the statistical differences were analyzed by the analysis of covariance (ANCOVA). The relationships between cumulative water consumption and cumulative dry weight indicated were evaluated by linear regressions through the origin. The statistical differences on the slopes of regression lines between two [CO_2_] environments were performed by ANCOVA and indicated the differences of WUE_plant_. The differences of [ABA]_xylem_ at a certain point between the two [CO_2_] concentrations were determined by Student’s *t*-test. In order to discriminate the means between the eight treatments, one-way ANOVA (Tukey’s test) was conducted to determine the significant differences. For each genotype, the effects of [CO_2_], drought stress and their interaction on the expression of each *PIP* and *OST1* were analyzed by two-way ANOVA. Differences between treatments were considered as significant when *p* < 0.05.

Principle component analysis (PCA) of *K*_leaf_, *K*_leaf_, and [ABA]_xylem_ and gene expression of all *PIP*s and *OST1* were performed individually for AC and *flacca* in R version 4.0.0 (R Core Team, 2020). In addition, a PCA plot of all parameters in both genotypes was produced to reveal differences between the two genotypes.

## Results

### Leaf Transpiration, Plant Water Consumption, and Water Relation Characteristics

**Figure 1:**
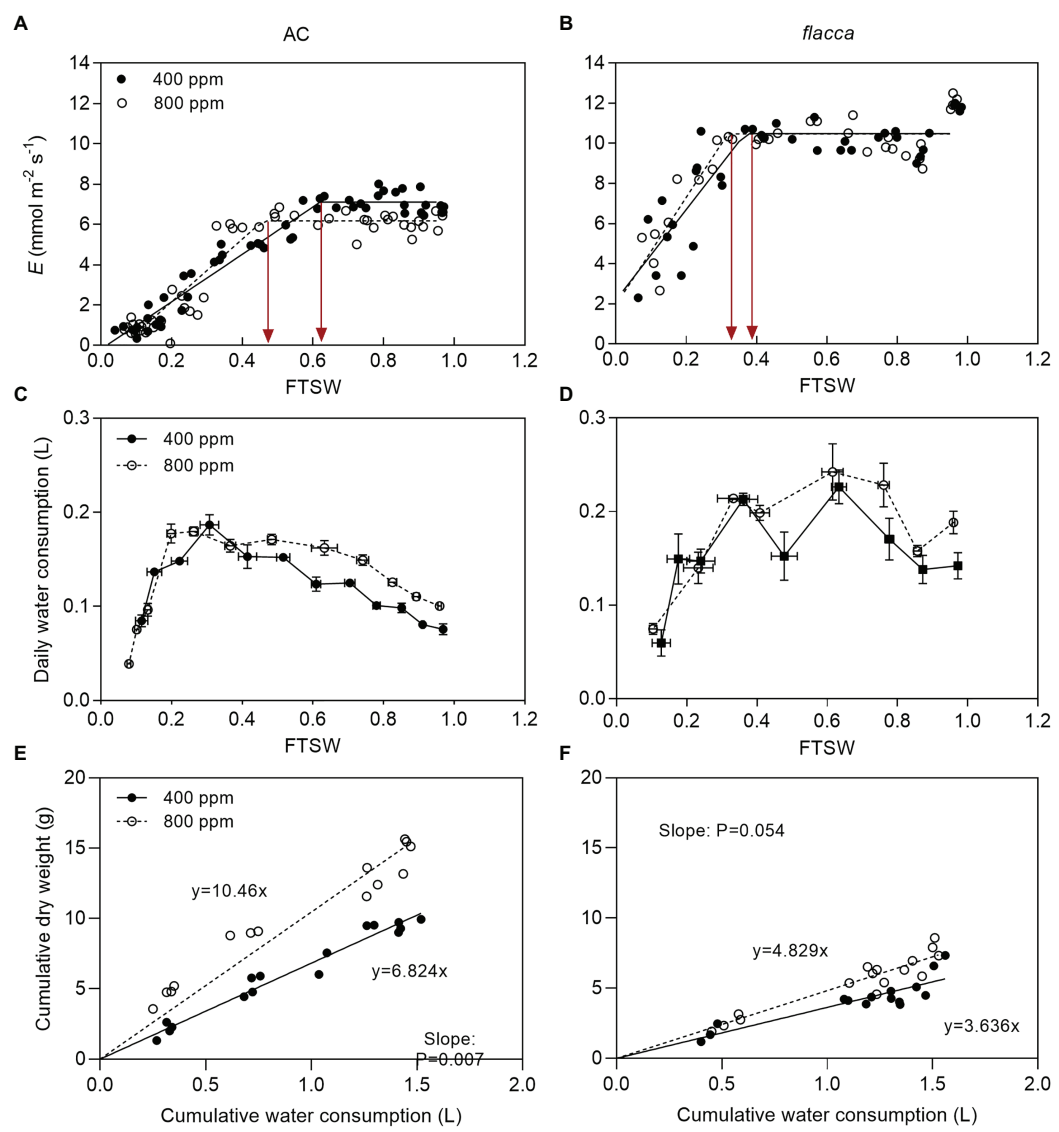
**(A,B)** changes of transpiration rate (*E*) of AC (total samples = 48, *N* = 4) and *flacca* (total samples = 32, *N* = 4) grown under ambient (400 ppm) and elevated (800 ppm) atmospheric CO_2_ concentrations during progressive soil drying. FTSW represents the fraction of transpirable soil water. Red lines represent FTSW thresholds, where *E* started to decline. **(C,D)** changes of daily water consumption of AC and *flacca* during progressive soil drying. Error bars indicate standard error of the means (S.E.; *N* = 3–4). **(E,F)** linear relationship between cumulative water consumption and dry weight of AC, and *flacca* during progressive soil drying. The slopes of the regression indicated plant water use efficiency (WUE_plant_). Closed circles indicate plants under 400 ppm CO_2_ concentration, and open circles indicate plants under 800 ppm CO_2_ concentration.

Before imposing drought stress, the maximum instantaneous transpiration rate (*E*) of AC was 32.2 and 41.0% lower under *a*[CO_2_] and *e*[CO_2_], respectively, compared to *flacca*. Moreover, the *E*_max_ of AC under *e*[CO_2_] was 13.1% lower than those under *a*[CO_2_]. During progressive soil drying, *E* of AC under *e*[CO_2_] started to decline at a lower FTSW threshold (C_E_) than that under *a*[CO_2_] (i.e., 0.46 vs. 0.62). While in *flacca*, there was no notable difference in *E*_max_ and C_E_ between the two [CO_2_] environments. In addition, the C_E_ of AC was higher than that of *flacca* under both *a*[CO_2_] and *e*[CO_2_] growth environments (i.e., 0.62 vs. 0.37 and 0.46 vs. 0.32, respectively; Figures 1A,B; Table 1). At the last harvest, in well-watered AC, depression on *E* by *e*[CO_2_] became less significant (Supplementary Figure S1A).

**Table 1:** Output of statistical analysis of parameters derived from the linear-plateau regression of transpiration rate (*E*), leaf and root hydraulic conductance (*K*_leaf_ and *K*_root_) of AC and *flacca* grown under ambient (400 ppm), and elevated (800 ppm) atmospheric CO_2_ concentrations response to the reduction in fraction of transpirable soil water (FTSW).

		400 ppm	800 ppm	Sig. level
AC	*E*_max_ (mmol m^−2^ s^−1^)	7.11 ± 0.12	6.18 ± 0.16	***
	C_E_	0.62 ± 0.10	0.46 ± 0.03	*
	*K*_leaf max_ (mmol m^−2^ s^−1^ MPa^−1^)	14.66 ± 0.41	10.22 ± 0.21	***
	C_kl_	0.47 ± 0.03	0.43 ± 0.08	ns
	*K*_root max_ (g cm^−2^ min^−1^ MPa^−1^)	1.11 ± 0.05	0.78 ± 0.03	***
	C_kr_	0.58 ± 0.04	0.51 ± 0.05	ns
*flacca*	*E*_max_ (mmol m^−2^ s^−1^)	10.49 ± 0.27	10.47 ± 0.22	ns
	C_E_	0.37 ± 0.04	0.32 ± 0.03	ns
	*K*_leaf max_ (mmol m^−2^ s^−1^ MPa^−1^)	11.35 ± 0.46	9.13 ± 0.29	***
	C_kl_	0.44 ± 0.06	0.42 ± 0.05	ns
	*K*_root max_ (g cm^−2^ min^−1^ MPa^−1^)	0.55 ± 0.04	0.47 ± 0.03	*
	C_kr_	0.44 ± 0.08	0.36 ± 0.05	ns

E_max,_ K_leaf max_, and K_root max_ indicated the initial values of those parameters when plants were not significantly affected by drought; C_E_, C_kl_, and C_kr_ indicated thresholds at which E, K_leaf_, and K_root_ started to decrease due to drought stress. The data are presented in Figure 1. Values are means ± SE. *, **, and *** indicate significant differences of the estimated parameters between the two [CO_2_] growth environments, and the two genotypes at *p* < 0.05, *p* < 0.01, and *p* < 0.001 level, respectively; ns denotes no significant difference.

Although *e*[CO_2_] decreased *E* in AC, it increased plant daily water consumption in both genotypes when FTSW ranged from 1.0 to ca. 0.4 (plants maintained the maximum *E* in this range of FTSW). However, when FTSW dropped below 0.4, there was no difference in daily water consumption of both genotypes (Figures 1C,D). Plant water use efficiency (WUE_plant_) was obtained from the slopes of the linear relationship between cumulative water consumption and cumulative dry weight. AC plants grown under *e*[CO_2_] had significantly greater WUE_plant_ than those under *a*[CO_2_] (*p* = 0.007), whereas no significant difference (*p* = 0.054) in WUE_plant_ for *flacca* was found between two [CO_2_] environments, though a clear tendency of increasing WUE_plant_ when grown at *e*[CO_2_] was observed for both genotypes (Figures 1E,F).

**Figure 2:**
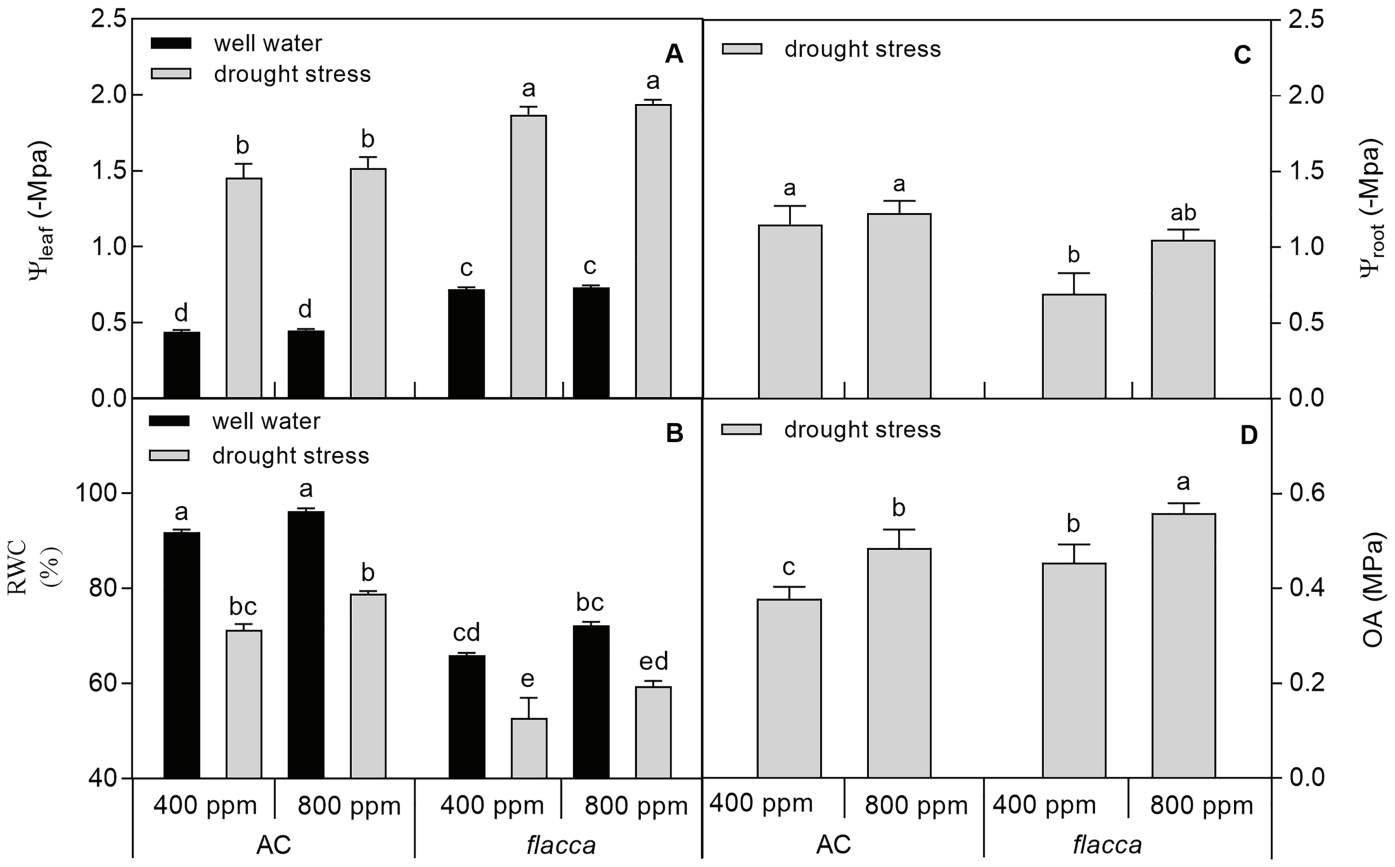
Leaf water potential (Ψ_leaf_; **A**), relative water content (RWC; **B**), root water potential (Ψ_root_; **C**), and osmotic adjustment (OA; **D**) of AC and *flacca* grown under ambient (400 ppm) and elevated (800 ppm) atmospheric CO_2_ concentrations after progressive soil drying. Black columns indicate plants grown under well-watered conditions, and gray columns indicate plants grown under drought stress. Different letters on the top of the columns indicate significant difference between the treatments by Tukey’s test at *p* < 0.05. Error bars indicate standard error of the means (SE; *N* = 4).

At the final harvest, drought stress decreased leaf water potential (Ψ_leaf_), root water potential (Ψ_root_), and leaf relative water content (RWC) in both AC and *flacca*, whereas *e*[CO_2_] had no influence on the two variables under both watering conditions. In addition, *e*[CO_2_] significantly increased osmotic adjustment (OA) in both AC and *flacca*, and AC had lower OA than *flacca* under both [CO_2_] environments (Figure 2).

### Leaf and Root Hydraulic Conductance

**Figure 3:**
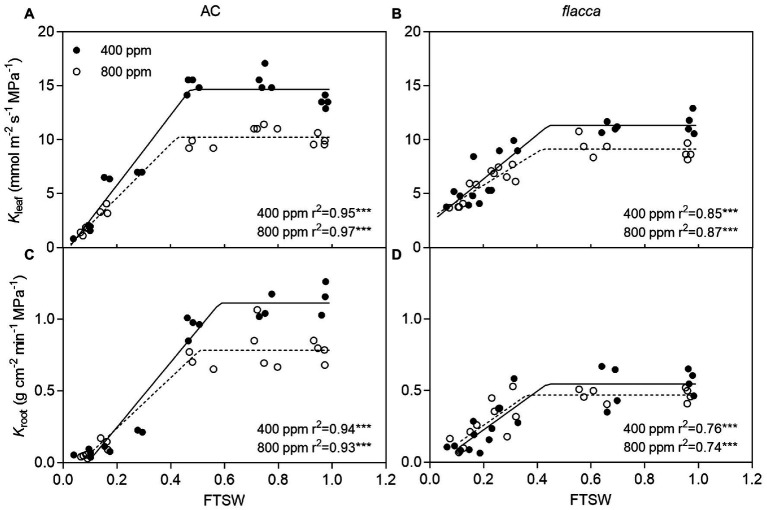
Changes of leaf and root hydraulic conductance (*K*_leaf_ and *K*_root_) of AC **(A,C)** and *flacca* (**B,D**; total samples = 17–20, *N* = 3–4) grown under ambient (400 ppm) and elevated (800 ppm) atmospheric CO_2_ concentrations during progressive soil drying. FTSW represents the fraction of transpirable soil water. Closed circles indicate plants under 400 ppm CO_2_ concentration, and open circles indicate plants under 800 ppm CO_2_ concentration.

When FTSW ranged from 1.0 to ca. 0.4, as the same with *E*, plants maintained the maximum *K*_leaf_ and *K*_root_, and AC had higher *K*_leaf max_ and *K*_root max_ than *flacca* especially under *a*[CO_2_]. Under *e*[CO_2_], AC had 30.29 and 29.73% lower *K*_leaf max_ and *K*_root max_ than *a*[CO_2_]-grown plants, respectively, whereas those effects were attenuated in *flacca* (19.56 and 14.55% lower *K*_leaf max_ and *K*_root max_ resulted by *e*[CO_2_]). During the course of progressive soil drying, in both genotypes, the FTSW thresholds (C_kl_ and C_kr_) did not respond to *e*[CO_2_] (Figure 3). At the last harvest, in well-watered AC, depression on *K*_leaf_ and *K*_root_ by *e*[CO_2_] was still observed (Supplementary Figures S1B,C).

### Xylem Sap ABA Concentration

**Figure 4:**
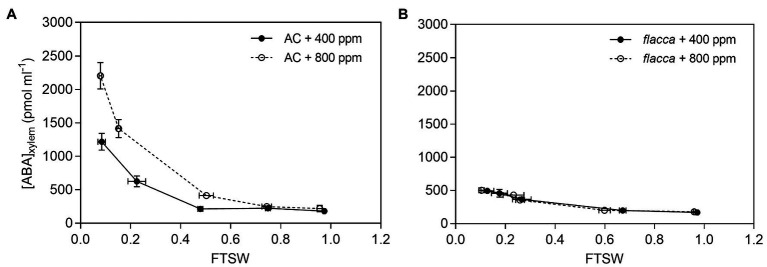
Trends of xylem sap ABA concentrations ([ABA]_xylem_) of AC **(A)** and *flacca* **(B)** grown under ambient (400 ppm) and elevated (800 ppm) atmospheric CO_2_ concentrations during progressive soil drying. Closed circles indicate plants under 400 ppm CO_2_ concentration, and open circles indicate plants under 800 ppm CO_2_ concentration. Error bars indicate standard error of the means (SE; *N* = 3–4).

The ABA concentration in xylem sap ([ABA]_xylem_) increased exponentially with the declining of FTSW in AC plants grown under both [CO_2_] environments, while the increase was more pronounced under *e*[CO_2_] than under *a*[CO_2_] and resulting in a greater [ABA]_xylem_ in the *e*[CO_2_] plants compared to *a*[CO_2_] plants during progressive soil drying. For *flacca*, [ABA]_xylem_ increased during soil drying and was identical between the two [CO_2_] environments, and remained significantly lower than those of the AC plants (Figure 4).

### Transcriptional Responses of Genes Encoding Plasma Membrane Intrinsic Proteins and Open Stomata1 Protein Kinase

**Figure 5:**
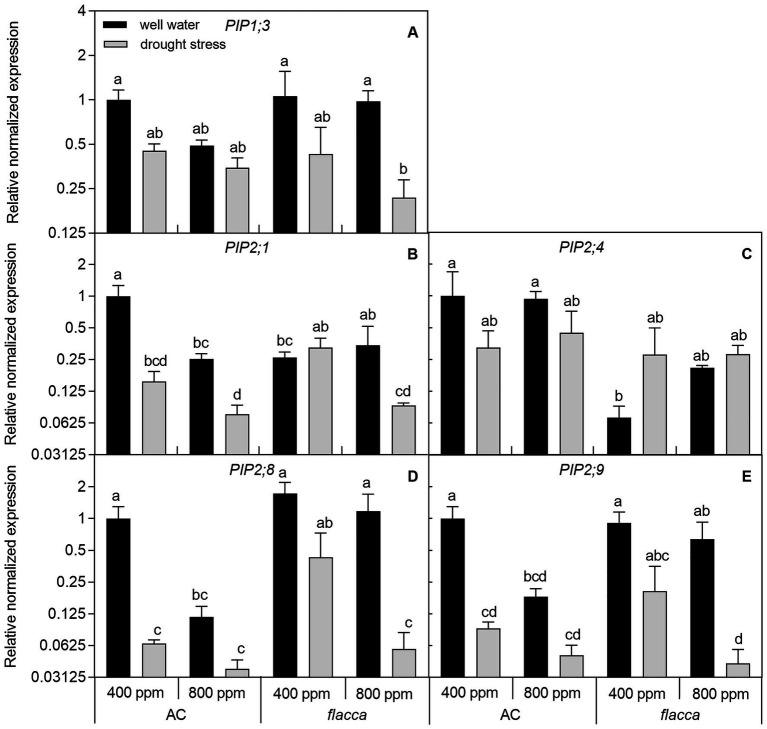
Relative expression levels of genes encoding aquaporin subfamily of the plasma membrane intrinsic proteins (PIPs; **A-E**) in leaves of AC and *flacca* grown under ambient (400 ppm) and elevated (800 ppm) atmospheric CO_2_ concentrations after progressive soil drying. Gray columns indicate plants grown under well-watered conditions, and white columns indicate plants grown under drought stress. Different letters on the top of the columns for each PIP gene indicate significant difference between the treatments by Tukey’s test at *p* < 0.05. Error bars indicate standard error of the means (SE; *N* = 4).

In leaves of well-watered AC plants, transcripts of four PIPs (*PIP1;3*, *PIP2;1*, *PIP2;8*, and *PIP2;9*) responded to *e*[CO_2_] with a 2–9-fold downregulation of expression levels, and those effects were absent in *flacca*. Moreover, transcripts of those same four PIPs were 2–15-fold downregulated by drought under *a*[CO_2_], and an interaction between [CO_2_] and drought stress was observed, showing that those downregulation became less significant under *e*[CO_2_]. In *flacca*, drought also downregulated the transcript levels of *PIP1;3*, *PIP2;8*, and *PIP2;9* with more than 2-fold change under both [CO_2_] environments. When comparing leaf PIP transcript levels between the two genotypes, AC showed higher expression levels of *PIP2;1* and *PIP2;4* than *flacca* under *a*[CO_2_]. In addition, within five PIPs, *PIP2;8* and *PIP2;9* were dramatically influenced by drought in both AC and *flacca* (Figure 5; Table 2).

**Table 2:** Results of two-way ANOVA test showing the statistical significance of the effects of [CO_2_] and drought stress (DS) on relative expression levels of genes encoding aquaporin subfamily of the plasma membrane intrinsic proteins (PIPs) and OPEN STAMATA 1 protein kinase (OST1) in leaves, and PIPs in roots.

		Leaf						Root				
		*PIP1;3*	*PIP2:1*	*PIP2;4*	*PIP2;8*	*PIP2;9*	*OST1*	*PIP1;3*	*PIP2:1*	*PIP2;4*	*PIP2;8*	*PIP2;9*
AC	[CO_2_]	**	*	ns	**	*	*	ns	**	ns	**	ns
	DS	**	**	ns	**	**	**	***	***	***	ns	ns
	[CO_2_] × DS	*	*	ns	*	*	ns	ns	ns	ns	**	*
*flacca*	[CO_2_]	ns	ns	ns	ns	ns	ns	***	***	**	***	***
	DS	*	ns	ns	**	**	*	***	***	***	***	***
	[CO_2_] × DS	ns	ns	ns	ns	ns	ns	*	**	ns	*	ns

**Figure 6:**
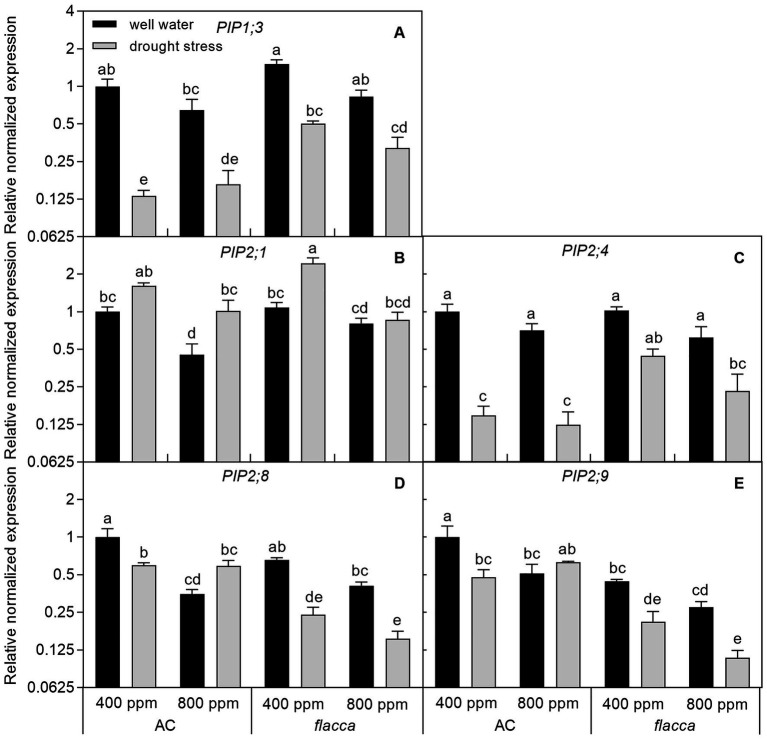
Relative expression levels of genes encoding the plasma membrane intrinsic proteins (PIPs) **(A-E)** in roots of AC and *flacca* grown under ambient (400 ppm) and elevated (800 ppm) atmospheric CO_2_ concentrations after progressive soil drying. Gray columns indicate plants grown under well-watered conditions, and white columns indicate plants grown under drought stress. Different letters on the top of the columns for each PIP indicate significant difference between the treatments by Tukey’s test at *p* < 0.05. Error bars indicate standard error of the means (SE; *N* = 4).

**Figure 7:**
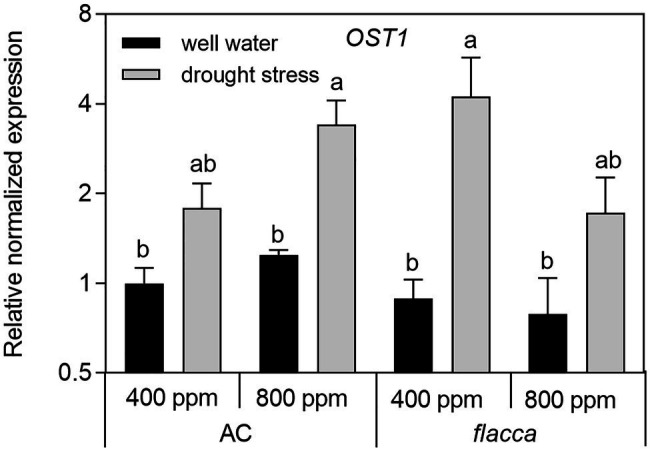
Relative expression levels of the gene encoding the OPEN STAMATA 1 protein kinase (OST1) in leaves of AC and *flacca* grown under ambient (400 ppm) and elevated (800 ppm) atmospheric CO_2_ concentrations after progressive soil drying. Gray columns indicate plants grown under well-watered conditions, and white columns indicate plants grown under drought stress. Different letters on the top of the columns indicate significant difference between the treatments by Tukey’s test at *p* < 0.05. Error bars indicate standard error of the means (SE; *N* = 4).

*, **, and *** indicate significant differences of the variables at *p* < 0.05, *p* < 0.01, and *p* < 0.001 level, respectively; ns denotes no significant difference. The data are presented in Figures 5–7.

Generally, *PIP* expression levels in AC roots were less sensitive to *e*[CO_2_] than the same PIPs in leaves. In well-watered AC plants, *PIP2;1*, *PIP2;8*, and *PIP2;9* showed indications of being 2–3-fold downregulated in response to *e*[CO_2_]. In *flacca*, all five *PIP*s responded to *e*[CO_2_], but downregulations of *PIP1;3*, *PIP2;1*, and *PIP2;8* expressions were only significant in stressed plants. In both genotypes, 2–8-fold downregulation of *PIP1;3* and *PIP2;4* were observed in response to drought under both [CO_2_] environments; whereas a slight upregulation of *PIP2;1* transcript level was observed in both AC and *flacca*. Interestingly, in AC plants, an interaction between [CO_2_] and drought stress on expression levels of *PIP2;8* and *PIP2;9* also existed, showing attenuated drought response under *e*[CO_2_]. When comparing root PIP expression levels between the two genotypes, the only expression of *PIP2;9* was found to be significantly different (Figure 6; Table 2).

In leaves of AC plants, the expression levels of *OST1* were slightly upregulated by *e*[CO_2_], and there was an interaction between [CO_2_] and drought stress, being more significant in stressed plants. Under drought stress, *OST1* transcripts were 2–5-fold upregulated in both AC and *flacca*. Moreover, there was no difference in *OST1* transcripts between the two genotypes (Figure 7).

### PCA Analysis of Hydraulic Conductance, Xylem Sap Aba Concentration, and Gene Expression

**Figure 8:**
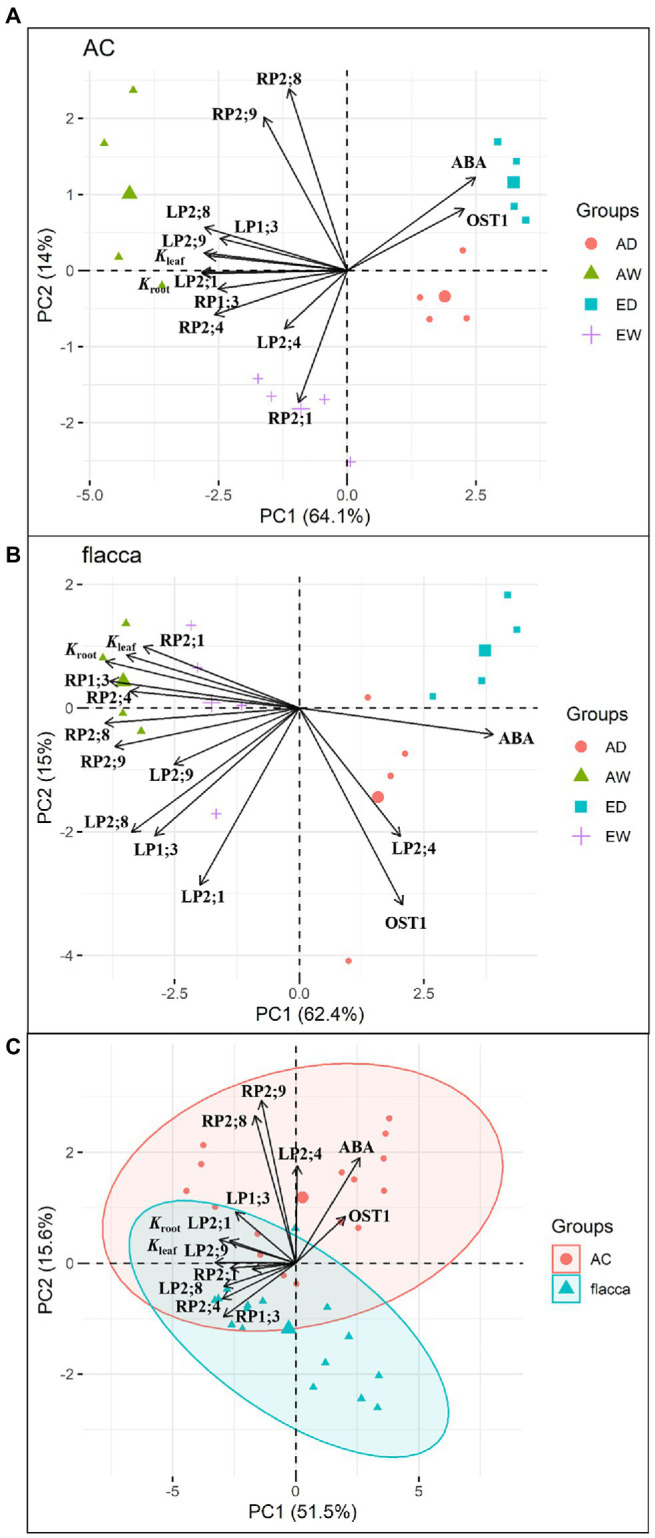
Principal component analysis of tomato leaf and root hydraulic conductance (*K*_leaf_ and *K*_root_), xylem sap ABA concentration (ABA), gene expression of five leaf *PIP*s (LP), root *PIP*s (RP) and *OST1* for **(A)** AC, **(B)** *flacca*, and **(C)** two genotypes grown under ambient (400 ppm) and elevated (800 ppm) atmospheric CO_2_ concentrations after progressive soil drying. AW: *a*[CO_2_] and well water; AD: *a*[CO_2_] and drought stress; EW, *e*[CO_2_] and well water; ED: *e*[CO_2_] and drought stress. The contribution of each PCA axis (PC1 and PC2) is indicated on the graph.

PCA plots of *K*_leaf_, *K*_leaf_, and [ABA]_xylem_ and gene expression of all leaf/root *PIP*s and *OST1* for AC and *flacca* were depicted in Figure 8. Overall, PCA showed clear group formation related to the [CO_2_] and watering treatments, and PC1 and PC2 axes explained 78.1 and 77.4% of the variation for AC and *flacca*, respectively. In both genotypes under both [CO_2_] (Figures 8A,B), the well water treatments were clustered in the same direction as the vector for the following parameters related to *K*_leaf_, *K*_leaf,_ and a majority of *PIP*s; the drought stress treatments were clustered in the same direction as the vector for *OST1* and ABA. However, in AC, *e*[CO_2_] showed a strong tendency with root *PIP2;1,* and ABA presented the smallest angle to *OST1*; in *flacca*, leaf *PIP2;4* was opposed by most *PIP*s and hydraulic conductance, and no covariation of ABA and *OST1* was observed. In addition, under well-water conditions, *a*[CO_2_] and *e*[CO_2_] treatments are separated on each side of the plot only in AC. In Figure 8C, PCA of all the parameters in two genotypes revealed that AC and *flacca* observations clustered toward the top and the bottom of the plot, respectively, and separation along PC2 confirmed the different responses in two genotypes regarding to ABA, *OST1*, leaf *PIP2;4*, root *PIP2;8* and *PIP2;9*.

## Discussion

### Elevated CO_2_ Improved Plant Water Status Under Progressive Soil Drying

There is a common understanding that *e*[CO_2_] can ameliorate the negative impacts of drought stress and enhance plant drought tolerance due to curtailed transpiration and enhanced carbon accumulation, hereby leading to an increase in WUE_plant_ (Robredo et al., 2007; van der Kooi et al., 2016; Li et al., 2020). Consistent with this, here we found that under mild drought stress, AC plants grown under *e*[CO_2_] had lower *E* and greater WUE_plant_ despite of an increased daily water consumption at the whole plant level (Figure 1). An increase in plant water consumption could be ascribed to a larger leaf area when grown under *e*[CO_2_], which has previously been reported by many researchers (Manea and Leishman, 2015; Temme et al., 2019). In *flacca*, it has been found that opened stomata resulted in faster photosynthetic induction at a cost of reduced WUE, which is the case in the present study (Kaiser et al., 2020). During progressive soil drying, it is notable that the FTSW threshold at which *E* started to decrease was retarded by *e*[CO_2_] only in AC but not in *flacca*, affirming our earlier findings that *e*[CO_2_] could delay stomatal response to drought in tomato plants in an ABA-dependent way (Wei et al., 2020). Kaiser et al. (2020) also reported that compared with wild-type, *flacca* lacked the ability to respond to CO_2_ or vapor pressure deficit in terms of *g*_s_. At last harvest, although Ψ_leaf_ and Ψ_root_ did not respond to *e*[CO_2_], slight effects of *e*[CO_2_] on RWC and OA were found in both AC and *flacca* (Figure 2). A high OA might be due to increased accumulation of solutes, which is considered to be associated with drought resistance (Jensen et al., 1996), and the accumulation of compatible solutes also could protect specific cellular functions and maintain leaf turgor (Johnson et al., 2002; Liu and Stützel, 2002). Therefore, plants grown under *e*[CO_2_] might possess improved drought resistance due to better water-holding capacity in leaves.

### Drought Stress Overrode the Effects of Elevated Co_2_ on Leaf and Root Hydraulic Conductance

Plant water balance under drought stress is controlled by a fine-tuned coordination between transpirational water loss and plant hydraulic conductance. As shown in Figure 1 and Figure 3, both AC and *flacca* plants maintained relatively high *E*, *K*_leaf_ and *K*_root_ until FTSW declined to a threshold value (ca. 0.4). Thereby, under mild drought stress conditions, plants still possessed favorable leaf and root hydraulic properties, which could be able to minimize the risk of excessive dehydration *via* a coordinated balance between leaf transpiration and water transport from root to leaf, consistent with the previous findings (Brodribb and Holbrook, 2004; Creek et al., 2018). Moreover, stomatal properties (e.g., stomatal size and density) are responsible for leaf water leak even when stomata are fully closed, which have a great impact on plant water use strategies (Machado et al., 2021). Dramatic higher stomatal density in *flacca* than in AC has been reported by Fang et al. (2019), which might explain the significant low *K*_root_ accompanied by still relatively high *E* and *K*_leaf_ in stressed *flacca*, representing an extensive hydraulic failure and dysfunction in the regulation of the plant water balance.

Although several studies reported that *e*[CO_2_] could decrease plant hydraulic conductance (Bunce, 1996; Robredo et al., 2007; Domec et al., 2009; Hao et al., 2018; Fang et al., 2019), there is no consensus about the roles of *K*_leaf_ and *K*_root_ in maintaining plant water status when combining *e*[CO_2_] and drought stress. As illustrated in Figure 3, under mild drought stress conditions (i.e., FTSW ranged from 1.0–0.4), *e*[CO_2_] decreased both *K*_leaf_ and *K*_root_, in two genotypes, though those depression was attenuated in *flacca*. However, at the last harvest, well-water *flacca* lacked the ability to respond to *e*[CO_2_] in terms of hydraulic conductance (Supplementary Figure S1), consistent with our the previous study (Fang et al., 2019). It should be noted that a slight increasing trend in [ABA]_xylem_ of *flacca* was still observed during progressive soil drying, which might contribute to the impact of *e*[CO_2_] on hydraulic conductance. Those contrasting results raised the possibility that different hydraulic responses existed under different intensities of drought stress. Overall, it is plausible that ABA is involved in the *e*[CO_2_]-induced changes in plant hydraulic conductance.

Under severe drought stress, i.e., FTSW < 0.4, *K*_leaf_ and *K*_root_ decreased sharply in both AC and *flacca* as well as *E* (Figure 3). Although it is widely believed that ABA could alter plant hydraulic properties (Parent et al., 2009; Rosales et al., 2019), our results demonstrated that hydraulic response to severe drought could be ABA-independent and might be attributed to decreased leaf turgor as reported in our previous study (Wei et al., 2020). Moreover, as shown in Figure 4, the increase of [ABA]_xylem_ in AC during progressive soil drying was more pronounced under *e*[CO_2_]. Although a study has shown that rapid ABA biosynthesis in angiosperms occurred in leaves rather than in root (Sussmilch et al., 2018), recently by using reciprocal grafting technique on AC and *flacca*, Li et al. (2018) found that long-distance ABA transport could affect foliar ABA concentrations under salinity but not control conditions. Moreover, when soil drying got severe, the release of root ABA into xylem could be attenuated due to the reduced water flow in roots (Liang et al., 1997), and decreased hydraulic conductance also might exert a drag on the delivery rates of ABA. Those results provide a clue concerning mechanisms of ABA metabolism and transport under *e*[CO_2_].

### PIPs Were Involved in the Modulation of Hydraulic Conductance by Elevated Co_2_ and Drought Stress

Several studies have demonstrated that the regulation of plant hydraulic conductance by ABA under drought stress is associated with modulating aquaporin activities (Parent et al., 2009; Dayer et al., 2017; Veselov et al., 2018), and *e*[CO_2_] could decrease the abundance of PIP1 and PIP2 proteins in both leaves and roots (Zaghdoud et al., 2013). In the present study, AC plants had higher transcript levels of genes encoding two and one out of five PIPs in leaves and roots, respectively, compared with those in *flacca* (Figures 5, 6). High transpiration demand could result in transcriptional upregulation of specific root PIPs, thus maintaining a favorable plant water status (Sakurai-Ishikawa et al., 2011). Therefore, discordance between high transpiration and low *PIP* levels in *flacca* might disturb plant hydraulic homeostasis, leading to desiccation of the leaves, which was the case in the present study. In addition, under well-watered conditions, *e*[CO_2_] had a more pronounced influence on leaf *PIP*s than root *PIP*s (Figures 5, 6), indicating that leaf *PIP*s might be more sensitive to the change in [CO_2_]. Our previous study has demonstrated that decreases in *K*_leaf_ and *K*_root_ of AC tomato plants under *e*[CO_2_] were associated with downregulation of *PIP*s, which was not the case for ABA-deficient mutant (Fang et al., 2019). However, here we found that the root *PIP*s of *flacca* still responded to *e*[CO_2_] though those effects were only shown in stressed plants, which was consistent with slight changes in hydraulic conductance of *flacca* (Figures 3, 6; Supplementary Figure S1; Table 2). In summary, *e*[CO_2_] could modulate plant hydraulic conductance *via* the regulation of leaf and root *PIP* transcript levels, where ABA had an obligate role but was dependent on the watering conditions.

At the end of drought treatment, there were dramatic decreases in *E*, Ψ_leaf_, Ψ_root_, RWC, *K*_leaf_, and *K*_root_, which accompanied by changes in transcriptional regulation of *PIP*s in both genotypes and [CO_2_] environments (Figures 1–3, 5, 6). The PCA in Figures 8A,B also confirmed that *e*[CO_2_] affected hydraulic conductance and the gene expression of *PIP*s only under well-watered but not drought-stressed conditions. Interestingly, the regulation of leaf *PIP* expression levels by drought was more significant compared with those in roots. As the ability of plants to conserve water during drought stress involves timely and sufficient downregulation of specific AQPs (Zupin et al., 2017), leaf *PIP*s might be more sensitive in their response to severe drought stress. In roots of both AC and *flacca*, the transcript levels of four out of five *PIP*s were downregulated by drought stress (Figure 6), which is in accordance with the previous studies (Shekoofa and Sinclair, 2018). Interestingly, in AC plants grown under *e*[CO_2_], the expression of *PIP2;8* and *PIP2;9* were not affected by drought, indicating that *e*[CO_2_] might disturb the ABA-mediated response of some root PIPs to drought stress, which merits further investigations. It should be noted that the expression of *PIP2;1* in roots was upregulated by drought in both AC and *flacca* (Figure 6), which represented a different expression pattern when compared to other root *PIP*s, indicating that PIP2;1 of tomato plants might play a crucial role in root water uptake especially under drought stress. In tobacco, NtPIP2;1 showed remarkable water transportability, though it was downregulated by drought stress in response to decreased *K*_root_ (Mahdieh et al., 2008; Secchi et al., 2016). Root PIPs and root anatomical properties were both correlated with hydraulic traits, and there is evidence that the contribution of root PIPs to *K*_root_ was enhanced by drought stress (up to 85%; Grondin et al., 2016). Therefore, a large genetic diversity for AQPs expression and root anatomy among various species might exit, and more research is necessary to underpin the multiple functions related to hydraulic traits. It is noteworthy to mention that here we only studied the leaf and root *PIP* transcript abundance under severe drought, and the previous report has shown that the expression patterns of *PIP*s were varied under different water stress intensities (Galmés et al., 2007). Therefore, further investigations to reveal the coordination between hydraulic conductance and PIP activities under progressive soil drying are needed.

### ABA Was Not Obligatory in the OST1-Mediated Drought Response

OST1 is a crucial component in the CO_2_ signaling pathway, and it may accelerate CO_2_ permeability under high [CO_2_] conditions (Wang et al., 2015). Here, we found that a significant effect of *e*[CO_2_] on the expression of *OST1* only existed in AC, suggesting that the function of *OST1* in CO_2_ transport might require ABA involvement. Recent reports showed that OST1 and PIP2;1 could function together to accelerate both water transport and CO_2_ transport in guard cells (Grondin et al., 2015; Wang et al., 2015), though here we did not found an obvious correlation between leaf transcripts of *PIP2;1* and *OST1*. In addition, there was no difference in expression of *OST1* between AC and *flacca* under either treatment, though *flacca* plants had dramatically lower Ψ_leaf_ and RWC under both well-watered and drought-stressed conditions. In Figure 8, the results of PCA highlight a significant difference in drought response of two genotypes regarding to the covariation of ABA and *OST1*. Nevertheless, *OST1* showed an upregulation of transcript levels under drought stress in both AC and *flacca* under either [CO_2_] environments (Figure 7), suggesting that factors rather than ABA (e.g., osmotic potential, Yoshida et al., 2006) could directly regulate the OST1-mediated drought response.

## Conclusion

**Figure 9:**
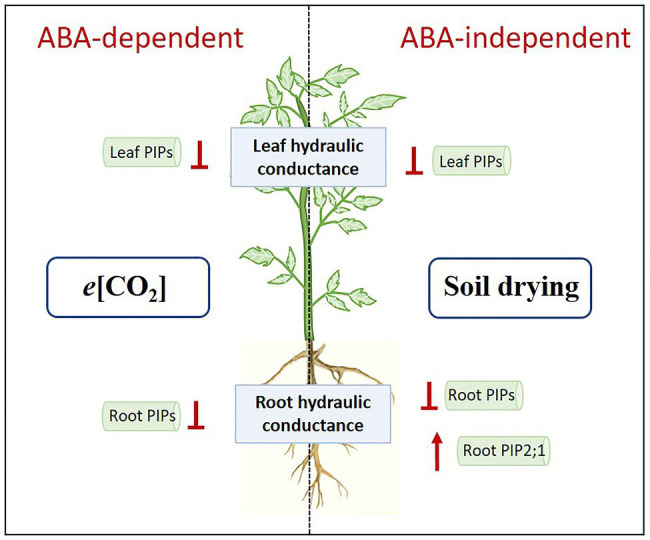
Hypothetical model of regulation of elevated CO_2_ (*e*[CO_2_]) and severe drought stress on plant water relations. Results of the present study indicate that *e*[CO_2_] could downregulate *PIP*s in leaves and roots, thus decreasing leaf and root hydraulic conductance, which was ABA-dependent. Meanwhile, severe drought stress could downregulate *PIP*s in leaves, which might be correlated with dramatic decrease in leaf hydraulic conductance. In roots, the regulation of *PIP*s by drought stress were varied: most of *PIP*s were downregulated; *PIP2;1* was upregulated. Those effects of drought stress were ABA-independent. When drought stress became severe, the effects of severe drought on plant hydraulic conductance could override those of *e*[CO_2_].

In this experiment, decreased *E*, *K*_leaf_, *K*_root_, increased WUE_plant_ and OA by *e*[CO_2_] could improve plant water status and contribute to drought resistance of tomato plants, but increased water demand might exaggerate plant vulnerability to severe drought stress. Under *e*[CO_2_], decreased *K*_leaf_ and *K*_root_ might be associated with downregulation of leaf and root *PIP*s, and ABA was required for this process. However, when plants were exposed to soil drying, the role of ABA became less important. Severe soil drying had a stronger impact on plant water relations than *e*[CO_2_], which directly modulated *E*, *K*_leaf_, and *K*_root_ in an ABA-independent way, coinciding with the changes in *PIP* transcript abundances (Figure 9). In addition, OST1 was also involved in drought response in the absence of ABA. A summary of these variable responses to *e*[CO_2_] and drought stress in two genotypes is shown in PCA (Figure 8). Our results also demonstrated that leaf PIPs were more sensitive to both drought and *e*[CO_2_] compared with those in roots, and *e*[CO_2_] might disturb ABA-mediated drought response where involving some PIPs.

## Data Availability Statement

The original contributions presented in the study are included in the article/Supplementary Material, further inquiries can be directed to the corresponding author.

## Author Contributions

SL, LF, and FL conceived the concept and carried out the experiment. JH contributed to the analysis of the results. SL wrote the manuscript with support from FL and JH. FL supervised the project. All authors contributed to the article and approved the submitted version.

### Conflict of Interest

The authors declare that the research was conducted in the absence of any commercial or financial relationships that could be construed as a potential conflict of interest.
