# Supplementary material for: Elevated CO2 Modulates Plant Hydraulic Conductance Through Regulation of PIPs Under Progressive Soil Drying in Tomato Plants
Source: Front Plant Sci. 2021 May 28;12:666066. doi: 10.3389/fpls.2021.666066 (PMC8218578; doi:10.3389/fpls.2021.666066)
Supplement: Supplementary file 1 [file Data_Sheet_1.docx]

## Supplementary data

**Figure S1.** Transpiration rate (*E*; **A**), leaf and root hydraulic conductance (*K*_leaf_; **B**, and *K*_root_; **C**) of well-watered AC and *flacca* grown under ambient (400 ppm) and elevated (800 ppm) atmospheric CO_2_ concentrations after progressive soil drying. Different letters on the top of the columns indicate significant difference between the treatments by Tukey’s test at P < 0.05. Error bars indicate standard error of the means (S.E.) (N=4).

**Table S1.** *PIP*, *OST1* and reference gene specific settings in quantitative real time PCR (RT-qPCR) runs and analyses.

**Figure S1**

**
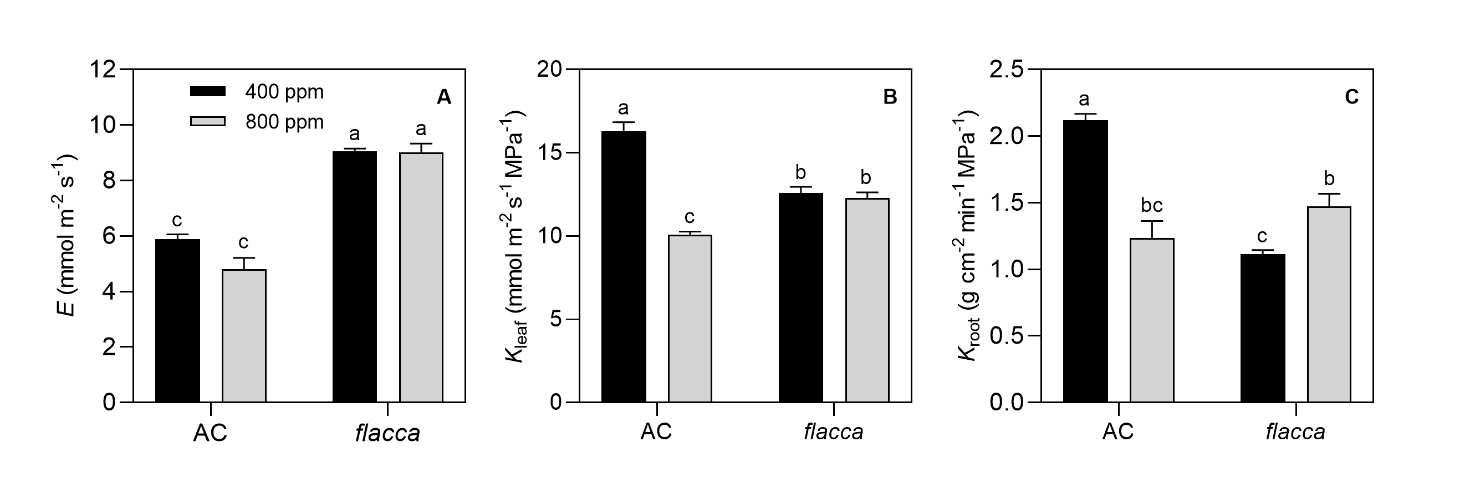
**

**Table S1**

| Target  gene | Temperature in RT-qPCR (°C) | Primer pair performance | | Sequence |
| --- | --- | --- | --- | --- |
|  |  | Efficiency | R^2^ |  |
| *PIP1.3*^a^ | 60 | 94.7 | 0.998 | F: 5’- GGCTACCATTCCAATCACCG -3’ |
|  |  |  |  | R: 5’- ATGATGATAGTTCACCAGG -3’ |
| *PIP2.1*^a^ | 60 | 92.1 | 0.997 | F: 5’- GTGCTGCTGTTGTTTATGGACA -3’ |
|  |  |  |  | R: 5’- CATCCAACACAACTCTAACAAC -3’ |
| *PIP2.4*^a^ | 60 | 100.3 | 0.998 | F: 5’- CAATGGTGACAAGGCGTGG -3’ |
|  |  |  |  | R: 5’- GAAGGCGAATTCATAGGAT -3’ |
| *PIP2.8*^a^ | 61.9 | 95.6 | 0.997 | F: 5’- GGAGCTGCTGTTATTGCTGA -3’ |
|  |  |  |  | R: 5’- GCACAGATCCAAGGCTAAGA -3’ |
| *PIP2.9*^a^ | 61.9 | 95.3 | 0.997 | F: 5’- GCAATGGCAGCAGCAATATACCA -3’ |
|  |  |  |  | R: 5’- CGAAAGAGAATAGACCACCA -3’ |
| *OST1*^b^ | 60 | 92.2 | 0.996 | F: 5’-CAGTTTGAGGAGCCAGATCA-3’ |
|  |  |  |  | R: 5’-GTCATCGTCAATGTCCAAGC-3’ |
| *TIP4.1*^c^ | 60 | 100.7 | 0.998 | F: 5’-ATGGAGTTTTTGAGTCTTCTGC-3’ |
|  |  |  |  | R: 5’-GCTGCGTTTCTGGCTTAGG-3’ |
| *SAND*^c^ | 58.4 | 101.1 | 0.997 | F: 5’-TTGCTTGGAGGAACAGACG-3’ |
|  |  |  |  | R: 5’-GCAAACAGAACCCCTGAATC-3’ |
| *CAC*^c^ | 61.9 | 107.3 | 0.997 | F: 5’-CCTCCGTTGTGATGTAACTGG-3’ |
|  |  |  |  | R: 5’-ATTGGTGGAAAGTAACATCATCG-3’ |
| *EXPR*^c^ | 60 | 99.1 | 0.996 | F: 5’-GCTAAGAACGCTGGACCTAATG-3’ |
|  |  |  |  | R: 5’-TGGGTGTGCCTTTCTGAATG-3’ |

^a^Reuscher, S., Akiyama, M., Mori, C., Aoki, K., Shibata, D., Shiratake, K., 2013. Genome-wide identification and expression analysis of aquaporins in tomato. PLoS One 8, e79052. https://doi.org/10.1371/journal.pone.0079052

^b^ Shi, K., Li, X., Zhang, H., Zhang, G., Liu, Y., Zhou, Y., Xia, X., Chen, Z., Yu, J., 2015. Guard cell hydrogen peroxide and nitric oxide mediate elevated CO2-induced stomatal movement in tomato. New Phytol. 208, 342–353. https://doi.org/10.1111/nph.13621

^c^Expósito-Rodríguez, M., Borges, A.A., Borges-Pérez, A., Pérez, J.A., 2008. Selection of internal control genes for quantitative real-time RT-PCR studies during tomato development process. BMC Plant Biol. 8, 1–12. https://doi.org/10.1186/1471-2229-8-131
